# Estimating Absolute Configurational Entropies of Macromolecules: The Minimally Coupled Subspace Approach

**DOI:** 10.1371/journal.pone.0009179

**Published:** 2010-02-23

**Authors:** Ulf Hensen, Oliver F. Lange, Helmut Grubmüller

**Affiliations:** 1 Department of Theoretical and Computational Biophysics, Max-Planck Institute for Biophysical Chemistry, Göttingen, Germany; 2 Department of Biochemistry, University of Washington, Seattle, Washington, United States of America; German Cancer Research Center, Germany

## Abstract

We develop a general minimally coupled subspace approach (MCSA) to compute absolute entropies of macromolecules, such as proteins, from computer generated canonical ensembles. Our approach overcomes limitations of current estimates such as the quasi-harmonic approximation which neglects non-linear and higher-order correlations as well as multi-minima characteristics of protein energy landscapes. Here, Full Correlation Analysis, adaptive kernel density estimation, and mutual information expansions are combined and high accuracy is demonstrated for a number of test systems ranging from alkanes to a 14 residue peptide. We further computed the configurational entropy for the full 67-residue cofactor of the TATA box binding protein illustrating that MCSA yields improved results also for large macromolecular systems.

## Introduction

Entropies are key quantities in physics, chemistry, and biology. While free energy changes govern the direction of all chemical processes including reaction equilibria, entropy changes are the underlying driving forces of ligand binding, protein folding and other phenomena driven by hydrophobic effect. Traditionally calculating entropies from atomistic ensembles 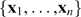 of 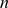 configurations 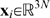 of a macromolecule of 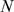 atoms remains notoriously difficult.

We here propose and apply a method for calculating configurational entropies
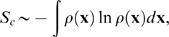
(1)where 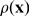 denotes the configurational probability density 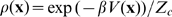 in the 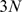 dimensional configurational space governed by the potential energy 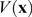 of the system. The fact that 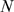 is usually on the order of several hundreds or thousands renders the evaluation of this integral quite challenging despite a number of successful attempts. [1]–[4] These broadly fall into three classes, (i) special-purpose perturbation type approaches, also known as thermodynamic integration [5], (ii) step-by-step reconstruction methods, in particular the scanning procedures introduced by Meirovitch [6], [7], (iii) direct approaches which analyse information readily available in standard equilibrium simulation trajectories [8]–[10].

While perturbation approaches provide relatively accurate free energy differences also for larger systems, accurate entropies are obtained only for smaller molecules. The main obstacle, which aggravates with system size, is the sampling problem, which severely limits the accuracy, in particular for explicit solvent models [2], [5].

The most widely used direct method is the quasi-harmonic approximation [8] (QH), which provides an upper limit to the configurational entropy in terms of 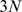 independent classical or quantum mechanical harmonic oscillators [9], [10], which is equivalent to approximating the configurational density 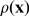 by a multi-variate Gaussian function,
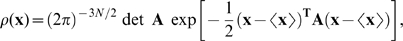
with 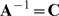 derived from the covariance matrix [9], [10] 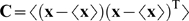. However, for macromolecules undergoing large conformational motions the entropy is likely to be considerably smaller than this QH upper limit due to coupling and anharmonicities and, in particular, due to the existence of multiple conformational states [11]–[14]. Indeed, for smaller systems such as di-saccharides [15] or lipids [16], or small subsets of larger proteins [17] significantly lower entropies than with QH were obtained by inclusion of anharmonicities [11]–[13], [18], [19] and pairwise correlation of QH modes [20].

## Results

### The MCSA Scheme

**Figure 1:**
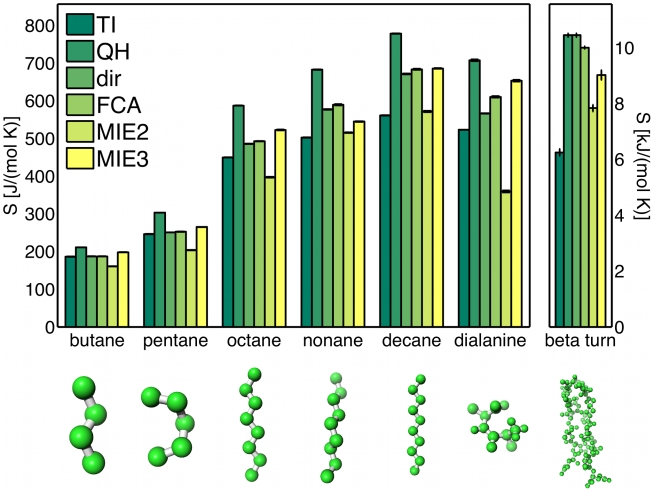
Entropy estimates for a set of small test systems. Five selected alkane systems, dialanine (left), and the C-terminal 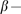
turn of Protein G (right, please note that here the units are kJ/(mol K)). Thermodynamic integration (TI), density estimates over the whole configurational space (dir), full correlation analyis with subsequent clustering and kernel density estimation (FCA), quasi-harmonic (QH) and mutual information expansion estimates of 2nd (MIE2) and 3rd (MIE3) order were obtained as described in the text.

Here we develop a direct method consisting of three building blocks. Results for small test systems will be presented during this introduction of the methodology to illustrate the effect of each building block. Figure 1 shows that indeed for various small test systems (alkanes, dialanine and a complete 14-residue 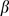-turn) the quasi-harmonic approximation severely overestimates the reference entropy. The reference values were obtained by thermodynamic integration (TI) gradually perturbing the systems towards an analytically tractable reference state consisting of non-interacting particles in harmonic wells, as described in methods and Refs. [21], [22]. Entropy estimates obtained for all test systems are also summarized in Table 1.

**Table 1:** Entropy estimates obtained for all systems.

System	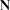	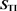	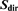	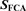	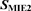	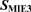	*clust*	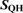
Butane	4	185 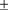 0.29	187 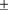 0.11	187 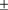 0.36	160 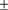 0.24	197 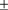 0.34	5	211 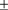 0.18
Pentane	5	245 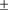 0.30	251 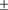 0.17	252 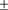 0.69	203 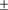 0.44	265 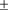 0.25	8	303 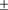 0.08
Hexane	6	307 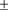 0.68	319 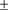 0.21	323 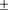 0.40	244 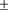 0.55	383 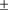 1.15	11	395 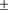 0.17
Heptane	7	388 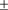 0.92	399 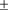 0.34	407 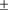 0.33	317 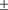 1.26	484 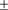 1.58	13	492 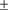 0.17
Octane	8	450 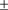 0.48	485 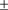 0.67	492 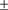 0.59	397 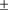 1.13	522 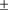 1.15	15	587 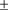 0.07
Nonane	9	502 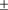 0.46	577 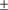 0.88	589 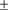 1.8	515 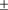 0.95	544 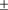 0.88	19	682 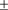 0.14
Decane	10	564 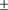 0.75	670 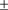 1.10	683 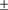 1.3	571 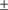 1.57	685 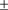 0.88	21	778 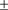 0.13
Dialanine	15	524 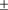 1.1	566 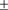 0.4	610 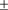 2.2	359 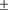 2.67	653 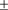 2.23	32	707 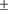 2.1
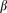 -turn	169	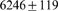	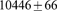	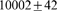	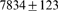	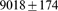	84–108	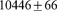
TBP cofactor	696	–	–	22250 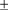 58	21543 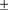 152	21853 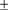 93	32–88	23226 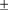 88
TBP complex	696	–	–	24918 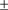 229	24371 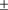 392	24514 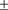 500	56–80	25880 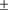 197

Alkane test systems butane to decane, dialanine, the 14-residue 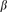-turn, as well as free and complexed TATA box binding protein (TBP) cofactor. 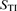: absolute configurational entropy obtained by TI (in J/(mol K)); 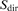: direct density estimate without clustering; 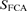: sum of density estimates after subspace clustering; 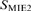 and 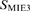 : Mutual information expansion estimates of 2nd (MIE2) and 3rd order (MIE3); 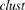: size of largest cluster; 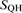: QH entropy estimate.

### Non-Parametric Density Estimation

As the first of the three building blocks of the methodology we recently introduced a non-parametric density estimation resting on adaptive anisotropic ellipsoidal kernels [21] that captures the configurational density in sufficient detail. Briefly, the configurational part of the entropy in a 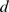-dimensional space is estimated from 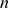 configurations according to
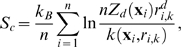
(2)where 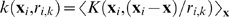 denotes the ensemble average of an adaptive anisotropic kernel function 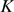, whose anisotropy and scaling 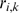 depends on the local density at point 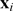, and whose 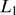-measure is denoted by 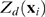. This formula simplifies to the well-known 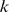-nearest neighbour entropy (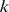-NN) by fixing the kernel function to an (isotropic) sphere whose radius 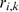 is chosen such that exactly 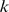 configurations are within the sphere centered at configuration 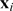. In this limiting case, 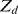 is the volume of the 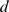-dimensional unit sphere. NN estimators in general are entirely non-parametric and, at a finite sample size 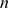, have minimal bias [23] in any given number of dimensions 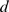. A major drawback, however, is the fact that due to the so-called ‘curse of dimensionality’ [24] simple 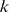-NN estimators are applicable for up to ten dimensional configurational spaces only [25]. In contrast, as can be seen in Fig. 1 (left, “dir”-bar), adaptive anisotropic kernels yield accurate results even for the 45-dimensional configurational space of dialanine. For the more than 500-dimensional configurational space of the 14-residue 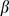-turn, however, the ‘curse of dimensionality’ [24] renders it impossible to improve on the quasi-harmonic approximation with direct density estimation alone (Fig. 1 right). Convergence properties and full technical details of this first MCSA module are discussed in Ref. [21].

### Generation of Minimally Coupled Subspaces

As the second building block of our method, we apply an entropy invariant transformation 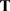 such that the usually highly coupled degrees of freedom separate into optimally uncoupled subspaces, each of which being sufficiently low-dimensional to render non-parametric density estimation applicable. As the most straightforward class of entropy invariant transformations, we consider here linear orthonormal transformations of the form 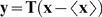 with 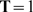. More general transformations are currently explored [26]. We apply Full Correlation Analysis (FCA) [27] which minimizes mutual information by considering
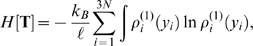
where 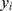 denote the components of 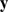 and 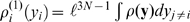 the 1-dimensional marginal density along 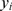. This procedure minimizes non-linear correlations of second and higher order [27] and therefore generalizes the principal component analysis (PCA) which only considers linear correlations of second order. For complex macromolecules, however, even for the optimal linear FCA transformation 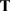, considerable non-linear correlations between several degrees of freedom will remain and cannot be neglected. To address this issue, the FCA modes are subsequently clustered according to the generalized correlation coefficient [25], [28]

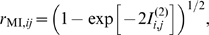
with the mutual information
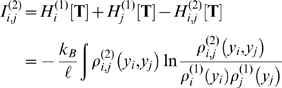
between components 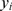 and 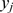. This is achieved by assigning mode indices 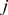 to 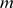 clusters 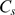 such that all modes with correlation coefficients larger than a certain threshold 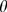 are assigned to the same cluster. This disjoint clustering defines an approximate factorization 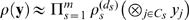 where 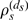 denotes the generalized 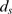-dimensional marginal density along 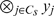. This factorization is approximate in the sense that for the entropy
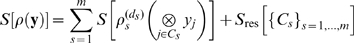
(3)the residual entropy 
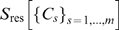
 is small.

Such approximate factorization, of course, neglects all inter-cluster correlations. These can be pairwise correlations, and thus are small 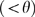 by construction, or higher-order correlations. For the latter we have to assume that they are also effectively eliminated by our threshold criterion. This assumption is supported by the observation that for the alkanes and for dialanine, with 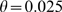, 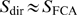 (cf. Fig. 1). Thus, our factorization yields accurate entropies and 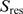 is indeed small.

### Mutual Information Expansions for Oversized Clusters

However, for the larger molecules considered here, the necessarily small threshold typically results in at least one cluster being too large for a sufficiently accurate density estimate (e.g., for the 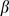-turn 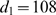). Accordingly, while our factorization still improves the entropy estimate (cf. Fig. 1), 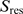 cannot be neglected anymore. The third building block of our method addresses this issue by subdividing each oversized cluster into 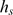 disjoint subclusters 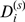 of sizes 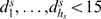, 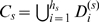, irrespective of the necessarily remaining strong correlations between these. The residual entropy contributions to the configurational entropy
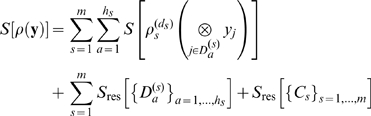
will be drastically increased due to non-neglegible intra-cluster contributions 
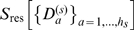
 from all subdivided clusters 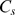, where we have omitted the argument 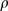 in the rightmost two terms for brevity. We here propose to compute each 
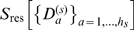
 via the mutual information expansion (MIE) as
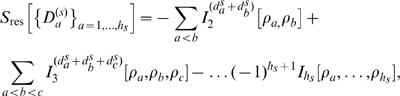
(4)where 
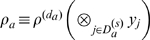
. Expanding the mutual information terms
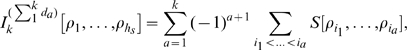
(5)up to second or third order, respectively, with the right-hand sum running over all possible permutations 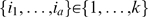, has proven sufficiently accurate in liquid state theory [29] and information theory [30], [31]. Indeed, for the 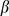-turn, inclusion of the remaining correlations via this expansion improved the entropy estimate (Fig. 1). For the other test systems 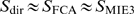. In contrast, for some of the test systems 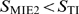, such that from our observations, 3rd order MIE provides a better estimate and an upper bound to the true entropy.

Applications of MIE to macro-molecular systems can be hampered by the curse of dimensionality and combinatorial explosion of the number of terms [32], [33]. In this work, the problem is circumvented by clustering into sufficiently high-dimensional (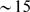) subspaces which minimizes residual inter-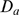 correlations and delays the onset of the combinatorial explosion. At the same time the subspaces are sufficiently small that even for the 3rd-order MIE no direct density estimates beyond the critical dimensionality of 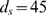 are required.

### TATA Box Binding Protein: Protein Test Case and Error Estimate

**Figure 2:**
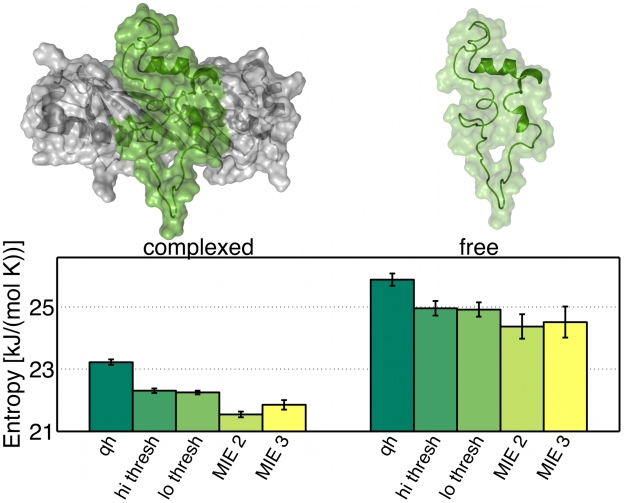
Entropy estimates for the TATA box binding protein (TBP) inhibitor in complex (left) and free (right). The following techniques are used: quasi-harmonic approximation (QH); FCA with subsequent density estimation using a high clustering threshold 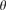 (hi thresh) or, respectively, a low threshold (lo thresh); mutual information expansion of order 2 (MIE2) or, respectively, of order 3 (MIE3). The displayed entropy estimates are averages over five independent simulations of 100 ns each, the error bars indicate standard deviations of the mean.

Together, these three building blocks enable one to calculate configurational entropies even for larger biomolecules. We considered the 67-residue TATA box binding protein (TBP, pdb code 1TBA) inhibitor in two different configurations; complexed (Fig. 2 top left) and free (Fig. 2 top right). To estimate the statistical error of MCSA and QH configurational entropy estimates, for both states five independent molecular dynamics (MD) simulations were carried out using the OPLS force-field [34] and the TIP4P explicit solvent model [35] (see methods section for full simulation details). Fig. 2 shows the results obtained by the five entropy estimation methods for both complexed (left) and free (right) inhibitor. All methods estimate the free cofactor's entropy to be significantly higher than that of the bound cofactor. As can be seen, for both complexed and free cofactor, QH yields the largest estimate. The first two MCSA modules combined (kernel density estimation on little correlated configurational subspaces obtained from FCA) already yield remarkably smaller estimates, irrespective of whether a high or a low clustering threshold 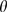 was chosen (hi thresh and low thresh in Fig. 2), i.e., chosing small but higher correlated subspaces or larger but lowly correlated subspaces provides similar estimates. Finally, employing all the three MCSA modules including MIE of 2nd (MIE2) and 3rd (MIE3) lowered the estimate again with, as before, the 2nd-order estimate being lower than the 3rd-order estimate.

The fact that the QH estimate is the largest in all cases corroborates the observations for the small test cases, and generally shows that MCSA yields improved estimates also for large macromolecules. Already the first two MCSA modules provide lower entropy estimates, even though relatively large configurational subspaces (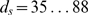, see Table 1) were obtained from FCA, which illustrates that indeed our kernel density estimator works accurately also for the complex high-dimensional configurational spaces spanned by proteins. Further, the fact that the clustering threshold did not affect the final estimate very much naturally reflects the fact that clustering with a high threshold yields small subspaces 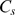 which are correlated, such that 
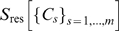
 in Eq. 3 is large, increasing our estimate 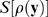. On the other hand, clustering with a small threshold gives rise to a small 
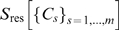
 but sparse sampling due to large 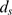 then entails higher 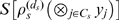, such that 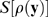 is also increased in this case. As expected, the third MCSA module, MIE, circumvents this problem and lowers the MCSA estimate further by 404 or 397 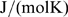 for the free and the complexed cofactor, respectively. The 2nd-order estimate is lower than the 3rd-order estimate in all cases, which shows that also for proteins the pair correlations are generally overestimated, and inclusion of 3rd-order correlations is indeed crucial.

The statistical errors are relatively small in all cases, but generally twice as large for the free than for the complexed cofactor. We attribute this observation to the larger inherent flexibility of the free state, and hence to insufficient molecular dynamics sampling. Consequently, the MIE error for the free cofactor is over three times larger than that of the the complex. Interestingly, the MIE estimate is slightly more affected with the error for the free cofactor being three- to fourfold as high as for the complex. Due to the high number of terms to be evaluated for the MIEs (Eq. 5), already small errors of each 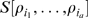 result in relatively large errors in 
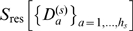
.

## Discussion

We have developed a minimally coupled subspace approach (MCSA) to estimate absolute macromolecular configurational entropies from structure ensembles which takes anharmonicities and higher-order correlations into account. The approach combines three building blocks which together allow one to calculate absolute entropies even for the highly complex configurational densities generated by the dynamics of biological macromolecules such as proteins. MCSA shares the versatility of the quasi-harmonic approach as it can be applied to unperturbed equilibrium trajectories while achieving the accuracy of special-purpose perturbation type methods. The effective dimension reduction provided by the Full Correlation Analysis allows for the application of mutual information expansions to large macromolecules. Further, the adaptive kernel non-parametric density estimation method developed for MCSA requires much weaker a-priori assumptions about the properties of the configurational densities than (quasi-)harmonic approaches. The method is applicable also to large macromolecules such as proteins. In this study, we showed that MCSA applied to the TATA box binding protein yielded significantly smaller and thus improved entropy estimates.

We note that here we focus at configurational entropies of the solute only, thus missing both the solvent as well as the solvent/solute parts. Using permutation reduction techniques [36], our method should be capable of capturing also these important contributions, which however lies outside the scope of the present work.

## Methods

### Thermodynamic Integration Reference Entropy

Absolute free energies for the test systems butane to decane, dialanine, and the ProteinG 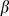-turn were calculated by thermodynamic integration (TI). Simulation parameters cf. below. The TI scheme we have chosen to obtain the Helmholtz free energy 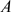 of the fully interacting particles consists of two phases. Harmonic position restraints with a force constant 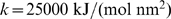 were slowly switched on for each atom in the first phase, and in the second phase all force-field components were gradually switched off. Within the second phase, the charges were switched off prior to the rest of the force field. After the second phase, the system consisted of non-interacting dummy particles with mass 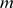 oscillating in their respective harmonic position restraint potentials, i.e.,
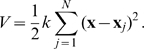



The free energy of this harmonic system can be obtained analytically,
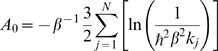
where 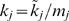 denotes the mass-weighted force constant. Hence, the thermodynamic integration yields the absolute free energy
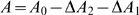
and the entropy by 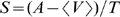, where 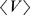 denotes the ensemble average of the potential energy.

For the TI between the systems given by 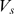 (start) and 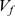 (end), 21 intermediate steps 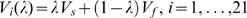 were used, and the intermediate values of 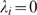, 1e-6, 5e-6, 1e-5, 5e-4, 1e-4, 1e-3, 1e-2, 2e-2, 3e-2, 5e-2, 7e-2, 9e-2, 0.1, 0.2, 0.3, 0.4, 0.5, 0.6, 0.7, 0.8, 0.9, 1 were distributed unevenly to obtain approximately balanced 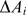 values. For each value of 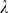 a trajectory of 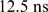 (alkanes and dialanine) or 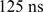 (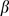-turn), respectively, was generated.

The error estimates of the TI reference entropies detailed in Table 1 were obtained via two ways for the alkane test systems and dialanine. First, by averaging over five independent simulations and, second, by performing blockwise averaging as derived in Ref. [37] over each of the 23 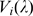 of each of these five trajectories. We found that the error estimates obtained by these two methods agree very well. Accordingly, for the 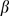-turn only the block averaging method was applied and the resulting error estimates are also given in Table 1.

### Molecular/Stochastic Dynamics Simulations

The test systems that were compared with a thermodynamic integration reference (butane to decane, dialanine, and the ProteinG 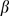-turn) were set up as follows. Force-field parameterizations were obtained from the Dundee Prodrug server [38] based on the GROMOS united-atom force field [39]. Stochastic Dynamics simulations were performed using the molecular simulations package GROMACS [40] in vacuo at 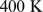 with friction constant 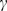 set to 10, dielectric constant 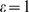, integration step size of 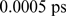 and no bond constraints. Positional restraints were applied to three adjacent terminal heavy atoms. To obtain MCSA error estimates, each of the simulations was carried out five times using different starting velocities. MCSA and QH entropy estimates were obtained from trajectories of lengths 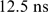 (alkanes and dialanine) or 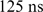 (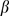-turn), respectively, i.e. the TI entropy references required 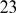 times as much computing time as MCSA and QH estimates.

The TATA box binding protein (TBP) complex (protein database entry 1TBA) was simulated using the OPLS all atom force field [34] in explicit TIP4P solvent [35] and periodic boundary conditions. NpT ensembles were simulated, with the protein and solvent coupled separately to a 300-K heat bath (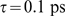). [41] The systems were isotropically coupled to a pressure bath at 1 bar (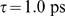) [41]. Application of the Lincs [42] and Settle [43] algorithms allowed for an integration time step of 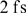. Short-range electrostatics and Lennard–Jones interactions were calculated within a cut-off of 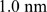, and the neighbour list was updated every 10 steps. The particle mesh Ewald (PME) method was used for the long-range electrostatic interactions [44], with a grid spacing of 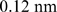. The free cofactor was simulated using the same parameters as above. The starting structure was obtained by removing the TBP from the X-ray structure of the complex and equilibrating for 2 ns. Entropy estimates and corresponding errors for both complexed and free cofactor were obtained from five trajectories of 200 ns length each.

### Mutual Information Expansions Implementation Details

#### Fill modes

**Figure 3:**
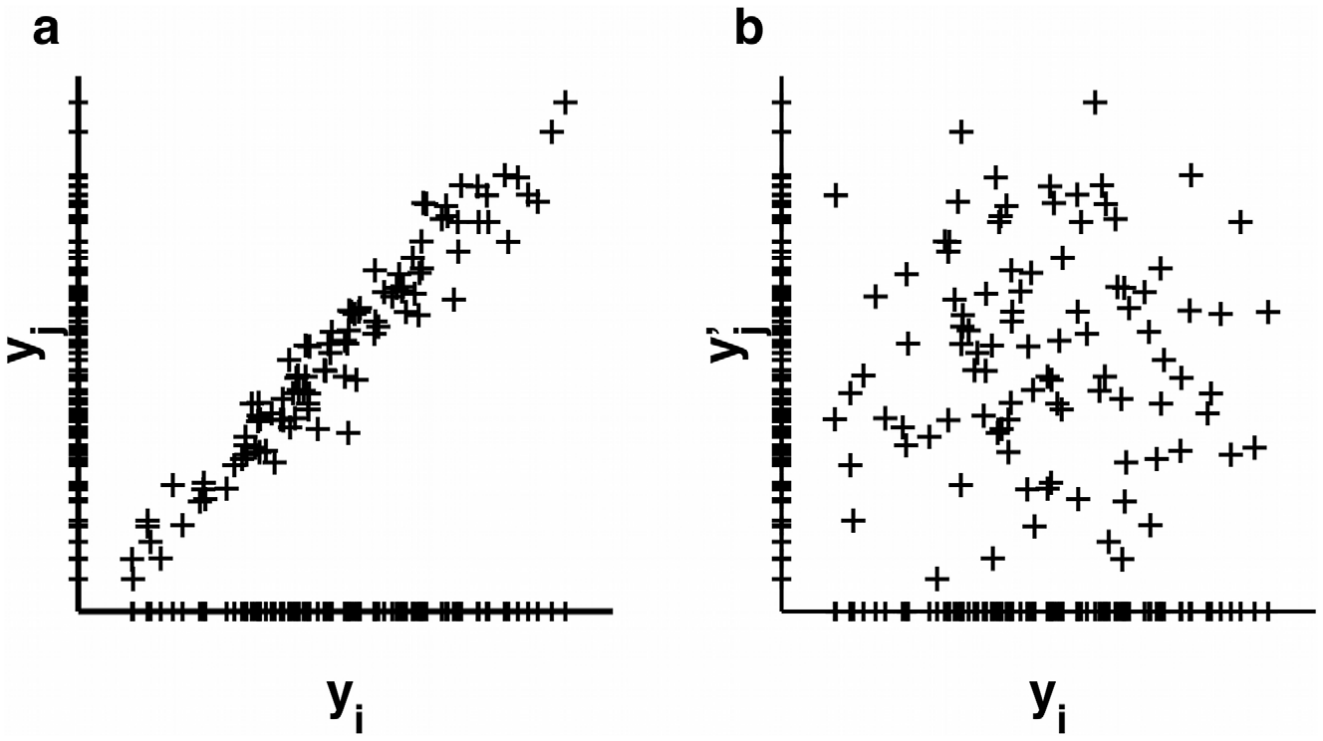
Principle of fill modes. a) Two arbitrarily correlated modes 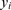 and 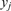 marginally distributed on the axes. Correlation is clearly visible from the 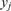-distributed 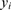. The joint distribution 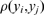 is more sparsely sampled than both marginal distributions. b) The 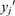-distributed 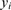 is decorrelated and has exactly as many sample points as the joint distribution in a), allowing precise computation of 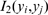.

Due to the moderate regularization assumptions, our adaptive kernel density estimator is sensitive to the sparse sampling problem whose effect is highly dependent on the dimensionality. To guarantee the same accuracy of all density estimates required for the computation of the correlation terms 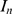 of Eq. 5 despite different dimensionality it is, thus, necessary to ensure the same local densities around points 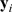 in different terms. This is normally not provided. The mutual information between two modes 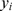 and 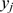,
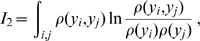
(6)contains differently well sampled terms in denominator and numerator, because the number of sampling points available to estimate 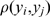 is only half the number of sampling points available for estimating the marginal densities 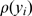 and 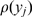 (see Fig. 3). The accuracy for the estimation of the marginal densities is, consequently, possibly higher than the joint estimate yielding an inaccurate correlation estimate. To overcome this problem, we devised the concept of fill modes. Accordingly, artificially decorrelated modes 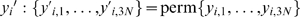 are created by permuting its components 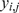, with 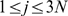. The marginal densities 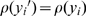 and 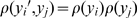, yielding a new expression for Eq. 6,
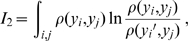
(7)where the product of the marginal densities 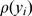 and 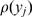 is now computed from the synthetically decorrelated joint distribution 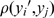, such that the same accuracy for the joint estimate is guaranteed as for the marginal estimates. Conducting this scheme on the 3rd order correlation function of three modes 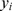, 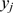 and 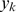,
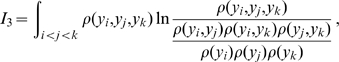
yields
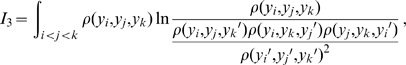
(8)where the pairwise joint distributions have been ‘filled up’ with permuted ‘fill modes’, as described above, e.g. 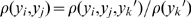.

#### Consistent dimensions

The sensitivity of the nearest-neigbour estimates, Eq. 2, towards the sparse sampling problem also affects the different terms of Eq. 5, which inevitably suffer from different sparse sampling problems if computed separately. Furthermore, a huge number of probability density distributions 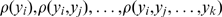 is computed more than once for the many instances of identical correlation terms appearing in that equation. Expanding over entropy terms rather than correlation terms, in contrast, yields
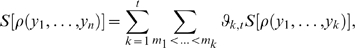
(9)where the first summation runs over different orders 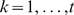 until truncation order 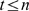. 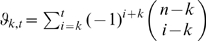 designates how many times a certain order appears and whether it needs to be added or subtracted, and the second sum over all 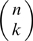 possible combinations 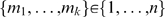. To guarantee the same estimation accuracy for all 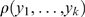 of Eq. 9, each term is filled up to truncation order 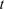 yielding 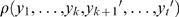. Under this modification, Eq. 9 reads
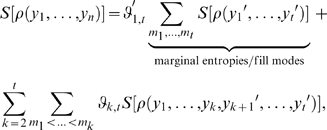
(10)with the number of marginal entropies,
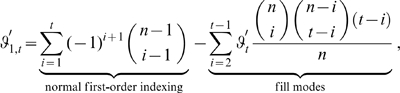
which depends on the fill mode weighting index
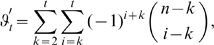
where, like above, primes indicate permuted entries.
